# Liver Cancer in Nepal

**DOI:** 10.5005/jp-journals-10018-1261

**Published:** 2018-05-01

**Authors:** Ananta Shrestha

**Affiliations:** Department of Hepatology, Liver Foundation Nepal, Kathmandu, Nepal

**Keywords:** Advanced stage, Hepatocellular carcinoma, Nepal, Treatment modalities.

## Abstract

Hepatocellular carcinoma (HCC) is highly incidental in South Asian countries. Nepal, however, has low incidence for HCC owing to low prevalence for hepatitis B virus (HBV) and hepatitis C virus (HCV) infections. Nepal lacked national cancer registry until 2003. Though there has been some effort in having one, the current registry incorporates twelve centers and may not properly represent the total cancer burden in the country. Serology for HBV and HCV is seen to be positive in nearly 25 to 30% and 5 to 10% of HCCs respectively. Clinical characteristics of HCCs in Nepal have been discussed in this mini-review and it features poor performance status and advanced stage at presentation, making only a small fraction of these subjects eligible for curative treatment options. Most of the standard treatment modalities are available in Nepal and appear to be reasonably affordable as compared with other developed nations.

**How to cite this article:** Shrestha A. Liver Cancer in Nepal. Euroasian J Hepato-Gastroenterol 2018;8(1):63-65.

## INTRODUCTION

Liver cancer is the 5th commonest cancer worldwide (sixth among men and eighth among women) and the second commonest cause of cancer-related death.^1^ South East Asia is known to have a very high incidence of HCC with age-standardized incidence rates (ASIRs) of 22.2 and 7.2 per 100,000 for men and women.^1^ The corresponding age-standardized mortality rates in South East Asia were 21.4 and 6.8 per 100,000. The relative incidence of HCC in different regions and countries within these regions goes along with the prevalence of viral hepatitis B and C.

## EPIDEMIOLOGY OF HCC IN NEPAL

Nepal is a developing South Asian country surrounded by India and China, which has intermediate and high prevalence of hepatitis B. Prevalence of HBV and HCV infection in Nepal is 0.9 and 0.4% respectively, making it a low-prevalence region in South Asia.^2,3^ Nepal did not have its cancer registry until 2003. Initially seven centers started contribution to form a cancer registry, and currently this has expanded up to 12 centers nationwide. Health care delivery including cancer care is fragmented with contribution from governmental and nongovernmental sectors. There is no integration and coordination between these sectors, and many health care providers do not notify cancer cases to the national cancer registry, making it less than representative. However, based on currently available data, crude incidence of liver cancer in Nepal is 0.9 and 0.8 per 100,000 in men and women respectively, and ASIRs are not known.^4^ The registry does not provide data on mortality rates associated with cancers. However, it was projected that age-adjusted mortality due to HCC in Nepal was 5.0 per 100,000 in 2000.^5^ Recent global disease burden estimation shows that age-adjusted annual mortality rates due to HCC is 2.8 per 100,000 in Nepal.^6^

## MORBIDITY ASSOCIATED WITH HCC

Liver cancer leads to a total of 73.1 years per 100,000 of healthy life lost or Disability-Adjusted Life Years (DALY).^6^ The age group most affected is 60 to 65 years in men and 65 to 70 years in men. At these age groups, DALY reaches 644.1 and 331 years per 100,000 in men and women respectively. As compared with other South Asian countries, the burden and impact of HCC in Nepal are relatively low.

**Table 1:** **Table 1:** Clinical characteristics of HCC patients in Nepal

*Characteristics*			
Mean age		59.3 ± 15.8 years	
Male:female		3:1	
Underlying cirrhosis		94%	
Child-Pugh class (A/B/C)		33/55/22%	
BCLC class (A/B/C/D)		16.7/5.6/22.2/55.6%	
Number of lesions (single/up to 3/multifocal)		33/10/56%	
Mean diameter of largest lesion (single/up to 3/multifocal)		6.28/6.35/11.82 cm	
ECOG performance score (0/1/2/3/4)		16.7/5.6/33.3/16.7/27.8%	

## ETIOLOGY OF LIVER CANCER IN NEPAL

Among 100 cases of HCCs studied between 1980 and 1987, based on serological assays, HBV infection was seen in 34.4% and HCV infection in 5.1%. In 2015 to 2017 HBV and HCV accounted for 25 to 30 and 9% respectively. However, when nucleic acid testing for HBV and HCV infections were done, HBV DNA was detected in 69% and HCV RNA was detected in 14% of HCCs in a study done in 2007.^7^

## CLINICAL PROFILE OF HCC PATIENTS IN NEPAL

The mean age of patients with liver cancer in Nepal was 40 years, with male to female ratio of 2:1 in an audit from 1980 to 1987. However, recent trends show mean age of 59 years (63.2 years in male and 45.7 years in female), with male to female ratio of 3:1.

In our audit, 94% of HCC occurred in the background of liver cirrhosis; 33% of HCC were Child-Pugh class I and 22% were Child-Pugh class III. Nearly 33% of HCCs had Eastern Cooperative Oncology Group (ECOG) performance status of >2.

At presentation, one-third of HCCs were single tumor, 55% of the HCCs were multifocal (>3 tumors). Even among those presenting as single tumor, mean diameter of tumor was 6.2 cm and in multifocal disease mean tumor diameter was 11.8 cm. Only 16.7% of HCCs were within Barcelona clinic liver cancer (BCLC) stage A at presentation, making only a small fraction of liver cancer curable in Nepal. Nearly half of the subjects were in BCLC stage D, eligible for supportive care alone The clinical characteristics of HCC patients in Nepal are summarized in Table 1.

## TREATMENT OF HCC IN NEPAL

Treatment modalities for HCC in Nepal along with their availability and approximate costs are summarized in Table 2.

**Table 2:** **Table 2:** Treatment modalities available for HCC and their cost in Nepal

*Treatment modalities*		*Availability*		*Cost*	
Percutaneous ethanol injection		Most of the tertiary centers		10-50 USD	
Radiofrequency/microwave/cryoablation		Not available		N/A	
Liver resection		Most of the tertiary centers		500-1000 USD	
TACE		Five centers		300-500 USD	
TARE		Not available		N/A	
Targeted therapy (sorafenib)		Available		300 USD/month	
Liver transplantation		Available (not done for HCC)		25,000-40,000 USD	

TACE: Transcatheter arterial chemoembolization; TARE: Transarterial radioembolization; USD: US dollar

**Graph 1:**
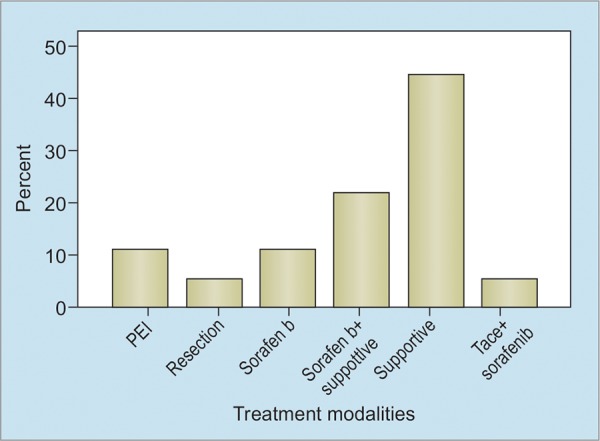
Treatment allocation for HCC patients in a single center in Nepal

## ELIGIBILITY FOR DIFFERENT TREATMENT MODALITIES

Nearly 15 to 20% of cases of HCC qualify for curative therapy including local ablation, resection, or transplant (Graph 1). Most of the cases (35-40%) present with locally advanced (BCLC-B and C) disease, making them eligible for locoregional therapy or targeted therapy. In rest (4050%) of the cases, presentation is usually at advanced stage (BCLC-D), and supportive therapy is all that can be offered.

## CONCLUSION

Nepal has one of the lowest incidence rates of HCC in SouthEast Asia. Hepatitis B and C are important preventable cause of HCC in Nepal, accounting for 25 to 30 and 10 to 12% of cases respectively. Most of the approved treatment modalities used for HCC are available in Nepal. The cost of treatment is reasonable and low compared with developed and other developing countries. However, most of the cases present in advanced stages, rendering them ineligible for curative therapeutic options. The need for adoption of proper screening strategy for high-risk groups (cirrhotics and chronic viral hepatitis) cannot be overemphasized, so as to increase the detection of early liver cancer amenable to cure.

## References

[1] Ferlay J, Soerjomataram I, Dikshit R, Eser S, Mathers C, Rebelo M, Parkin DM, Forman D, Bray F (2015). Cancer incidence and mortality worldwide: sources, methods and major patterns in GLOBOCAN 2012. Int J Cancer.

[2] Shrestha SM (1990). Seroepidemiology of hepatitis B in Nepal. J Commun Dis.

[3] Shrestha SM, Subedi NB, Shrestha S, Maharjan KG, Tsuda F, Okamoto H (1998). Epidemiology of hepatitis C virus infection in Nepal. Trop Gastroenterol.

[4] Poudel KK, Huang Z, Neupane PR, Steel R (2016). Changes in distribution of cancer incidence in Nepal from 2003 to 2013. Asian Pac J Cancer Prev.

[5] Lemon SM, Layden TJ, Seeff L, Suzuki H, Nishioka K, Mishiro S, Johnson L (2000). The 20th US-Japan joint hepatitis panel meeting. Hepatology.

[6] Liver Cancer in Nepal. Statistics on overall impact and specific effect on demographic groups. Nepal: Liver Cancer in Nepal; Available from:. http://global-disease-burden.healthgrove.com/l/30736/Liver-Cancer-in-Nepal.

[7] Shrestha SM, Shrestha S, Shrestha A, Tsuda F, Endo K, Takahashi M, Okamoto H (2007). High prevalence of hepatitis B virus infection and inferior vena cava obstruction among patients with liver cirrhosis or hepatocellular carcinoma in Nepal. J Gastroenterol Hepatol.

